# Impact of the COVID-19 pandemic on orthopaedic and traumatological care in Prague, the capital of the Czech Republic

**DOI:** 10.1371/journal.pone.0269164

**Published:** 2022-06-03

**Authors:** Petr Fulin, Matej Daniel, Jiri Walder, Dariusz Grzelecki, David Pokorny

**Affiliations:** 1 1st Department of Orthopaedics, First Faculty of Medicine of Charles University and Motol University Hospital, Prague, Czech Republic; 2 Department of Mechanics, Biomechanics, and Mechatronics, Faculty of Mechanical Engineering, Czech Technical University in Prague, Prague, Czech Republic; 3 Department of Orthopaedics and Rheumoorthopaedic, Centre of Postgraduate Medical Education, Otwock, Poland; Assiut University Faculty of Medicine, EGYPT

## Abstract

The coronavirus disease (COVID-19) has significantly affected society, especially healthcare systems worldwide. The aim of this retrospective study is to evaluate the impact of the COVID-19 pandemic on orthopaedic and trauma healthcare at the largest university hospital in the Czech Republic. The evaluated periods were in accordance with three waves of the disease and three respective lockdowns. To correlate the results, we evaluated the number of patients (inpatients and outpatients) treated in the same period in the last 3 years before the pandemic. The number of orthopaedic outpatients during the lockdown period decreased by 54.12% (p = 0.002), 42.88% (p <0.001), and 34.53% (p = 0.03) in the first, second, and third lockdowns, respectively. The number of elective surgeries decreased by 69.01% (p <0.001), 87.57% (p <0.001), and 74.89% (p = 0.007) and the number of acute surgeries decreased by 33.15% (p = 0.002), 37.46% (p <0.001), and 27.24% (p = 0.034) in the first, second, and third lockdowns, respectively. This study showed a significant reduction in the healthcare of orthopaedic and trauma patients owing to the COVID-19 pandemic and emphasised the shortcomings of the healthcare system. In our study, there was a reduction in both outpatient (reduction of care by 24–54%) and inpatient care. The elective surgeries were reduced by 69–87% during different lockdown periods compared with the reference period. Based on the results of this study, we can formulate organisational measures to mitigate the impact of the pandemic on orthopaedic healthcare. Organisational procedures created based on acquired data and experience should maximise the bed capacity of the workplace and work efficiency with regard to the safety of medical staff.

## Introduction

At the end of 2019, an unknown disease caused by the severe acute respiratory syndrome coronavirus (SARS-CoV-2) virus was discovered. The number of infections and deaths increased sharply in early 2020, and the disease began to spread worldwide [1]. Concerns about this unknown disease and its consequences led to the introduction of massive epidemiological measures in the form of lockdowns in many countries around the world [2–7]. There have been massive restrictions on services, operation of shops, the economy, and the free movement of people between and within countries [2–7]. Healthcare systems around the world were not prepared for such situations. These events led to a significant reduction in the care of orthopaedic patients between 2020 and 2021, and we are still struggling with the consequences of limited care.

In the Czech Republic, the government announced massive epidemiological restrictions during the first wave of the disease for the first time on 12^th^ March 2020 despite the relatively small number of cases (compared to the second and third waves of the disease) [8, 9]. During the first wave of the disease, the government did not restrict the care of orthopaedic and trauma patients. Northern Italy was the region that was most affected at the beginning of the pandemic [10, 11]. With the increase in the number of cases and mortality rate, this was an exemplary case of disease severity. Other countries in Europe and around the world began to introduce lockdowns even with relatively small number of patients. In many cases, government epidemiological measures were unclear. Concurrently, there was an acute lack of protective equipment (especially masks and respirators) [10, 11]. Consequently, there was a significant reduction in healthcare costs. Patients cancelled planned surgeries and outpatient treatment on a mass scale because of the fear of infection. This period lasted from 12^th^ March 2020 to 17^th^ May 2020 (67 days) and is referred to as the first lockdown [8, 9].

The period from 7^th^ October 2020 to 22^th^ November 2020 (47 days) is referred to as the second lockdown [8, 9]. There was a reduction in services and movement of people, closure of schools, and reduction of health care. The increase in the number of patients requiring inpatient care led to the closure of some departments and their transformation into COVID-19 units. Specifically, elective surgery was affected by being significantly reduced owing to capacity reasons (lack of beds and medical staff). Outpatient care for orthopaedic patients was also limited. The overall state of healthcare was also complicated by the lack of staff due to COVID-19. The period from 7^th^ December 2020 to 11^th^ April 2021 (126 days) is referred to as the third lockdown, and it had the same conditions prevailing as the second lockdown. In addition, this period was accompanied by legislative government restrictions on elective surgeries. During the second and third lockdowns, the government declared a state of emergency due to the COVID-19 pandemic. At this time, the compensation mechanisms were partially implemented. The planned orthopaedic examinations and regular control were postponed without compensation, and hospital visits were recommended only to patients with acute health problems. Owing to the lack of medical staff, virtual clinics or telemedicine methods were not implemented. Patients with acute trauma were treated under strict hygiene measures in full care. This study aimed to evaluate the impact of the COVID-19 pandemic on the pattern of surgical and outpatient orthopaedic and trauma care at the largest university hospital in the Czech Republic.

## Materials and methods

This was an descriptive retrospective case series study. This study evaluated the number of treated orthopaedic and trauma patients in the largest university hospital in the Czech Republic, the 1st Department of Orthopaedics of the First Faculty of Medicine, Charles University, and the Motol University Hospital in Prague during the pandemic of COVID-19. The Motol University Hospital has 2199 beds and over 1 million outpatients per year. The 1st Department of Orthopaedics has 128 beds for adult elective, oncology, and trauma surgeries. The data were obtained from the hospital information system of the Motol University Hospital and from the available epidemiological data of the Ministry of Health of the Czech Republic [8]. The evaluated periods were in accordance with the three waves of disease and the respective three lockdowns (government-ordered restrictions on services, people’s movements, and healthcare).

We evaluated the number of outpatients in the orthopaedic and trauma department of the clinic, the number of urgent and planned admissions for the surgery, and the spectrum of injuries to the musculoskeletal system. Outpatient trauma patients were divided into two groups. Emergency trauma patients were those with recent injuries. Control trauma outpatients were patients who came for control of an already treated injury (control radiography, wound control, etc.). These groups were divided according to the number of recent injuries.

To correlate the results, we evaluated the number of patients treated in the same period in the last 3 years prior to the pandemic.

Polytraumas admitted to the anaesthesiology and resuscitation department and severe spinal traumas (treated at specialised clinics) were not included in the evaluation. Owing to the fact that the clinic deals only with the treatment of adult patients, the care of paediatric patients (i.e. up to 18 years of age) was not included in the evaluation.

The study was approved by the University Hospital Ethics Committee under EC number 1685/20, titled ‘Monitoring the number of treatments for orthopaedic patients during the COVID-19 pandemic’. Because it was a retrospective evaluation of anonymised data, written consent to participate in the study was not required. The data analysis was in accordance with legislative standards and general data protection regulations.

The results were statistically evaluated using the R statistical program version 4.0.4. The normality of the data from the reference period was assessed using the Shapiro-Wilk test. The mean values of the patient numbers determined from the reference period were compared with the single values obtained during the pandemic using a one-sample t-test. Differences were considered statistically significant at p <0.01.

## Results

**Fig 1:**
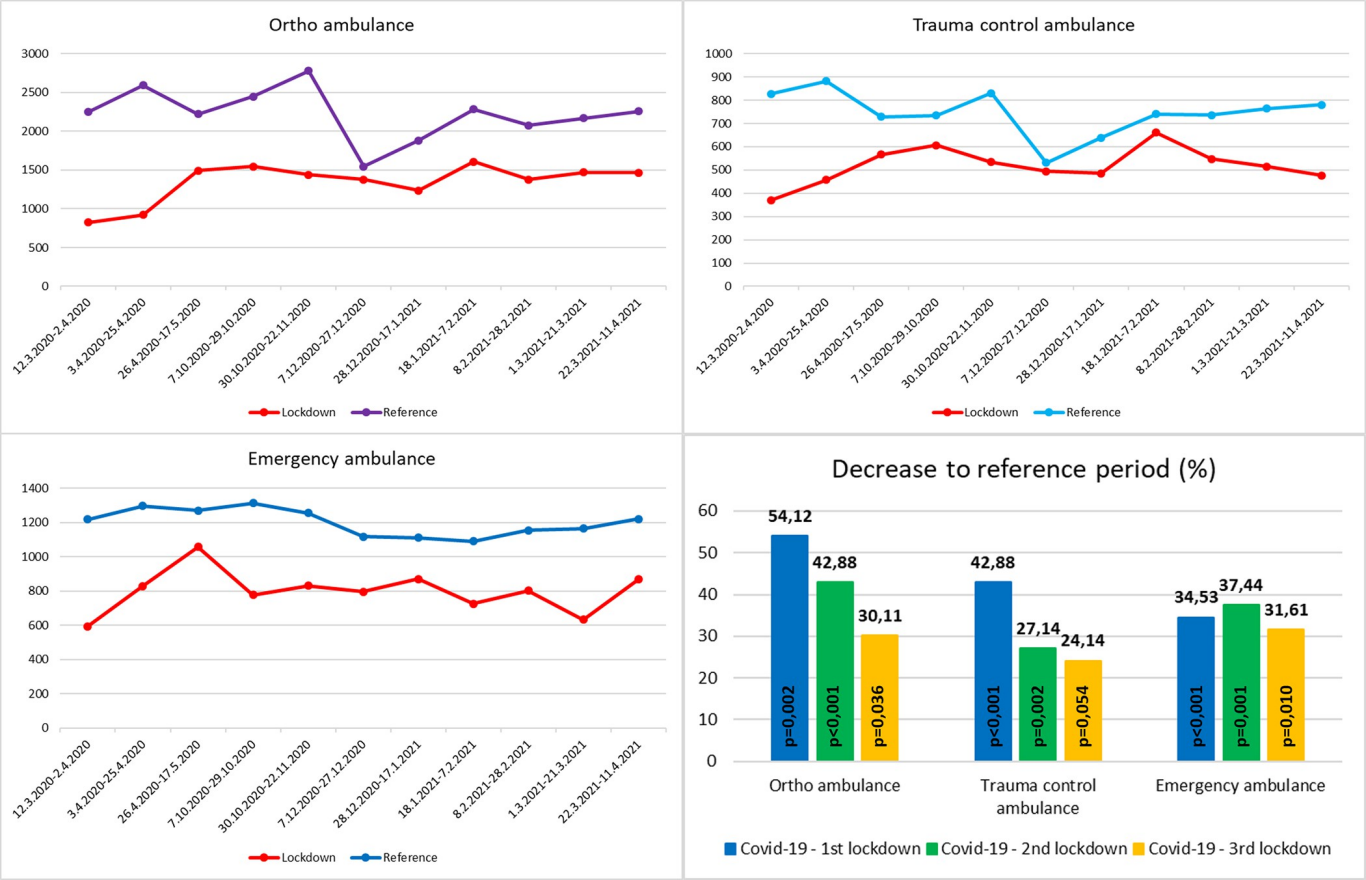
Overview of outpatients. Numbers of treated outpatients (y-axis) at individual ambulances during the lockdown period and the reference period. Percentage decrease (y-axis) in the number of treated outpatients in individual lockdowns compared to the reference period at the bottom right. For the reference period, the average of the previous 3 years before the pandemic is calculated. The p value indicates the statistical significance of the decrease.

Fig 1 shows an overview of the number of orthopaedic outpatients, control trauma outpatients, and emergency trauma outpatients in the lockdown and reference periods. The number of outpatients in the reference period was calculated as the average of the last 3 years before the pandemic (Fig 1). The number of orthopaedic outpatients during the lockdown period decreased by 54.12% (p = 0.002), 42.88% (p <0.001), and 34.53% (p = 0.03) in the first, second and third lockdowns, respectively, compared to the reference period of the previous 3 years. The number of control trauma outpatients during the lockdown period decreased by 42.88% (p <0.001), 27.14% (p = 0.002), and 37.44% (p = 0.05) in the first, second and third lockdowns, respectively compared with the reference period of the previous 3 years. The number of emergency trauma outpatients during the lockdown period decreased by 30.11% (p <0.001), 24.14% (p = 0.001), and 31.61% (p = 0.01) in the first, second and third lockdowns, respectively, compared to the reference period of the previous 3 years (Fig 1). During the entire lockdown period, 29,249 outpatients were treated at orthopaedic and trauma clinics, which was 16,659 patients lower in number than that in the reference period. Due to the length of the lockdown (240 days in total), there was a decrease of 69.41 outpatients per day (including weekends and holidays).

**Fig 2:**
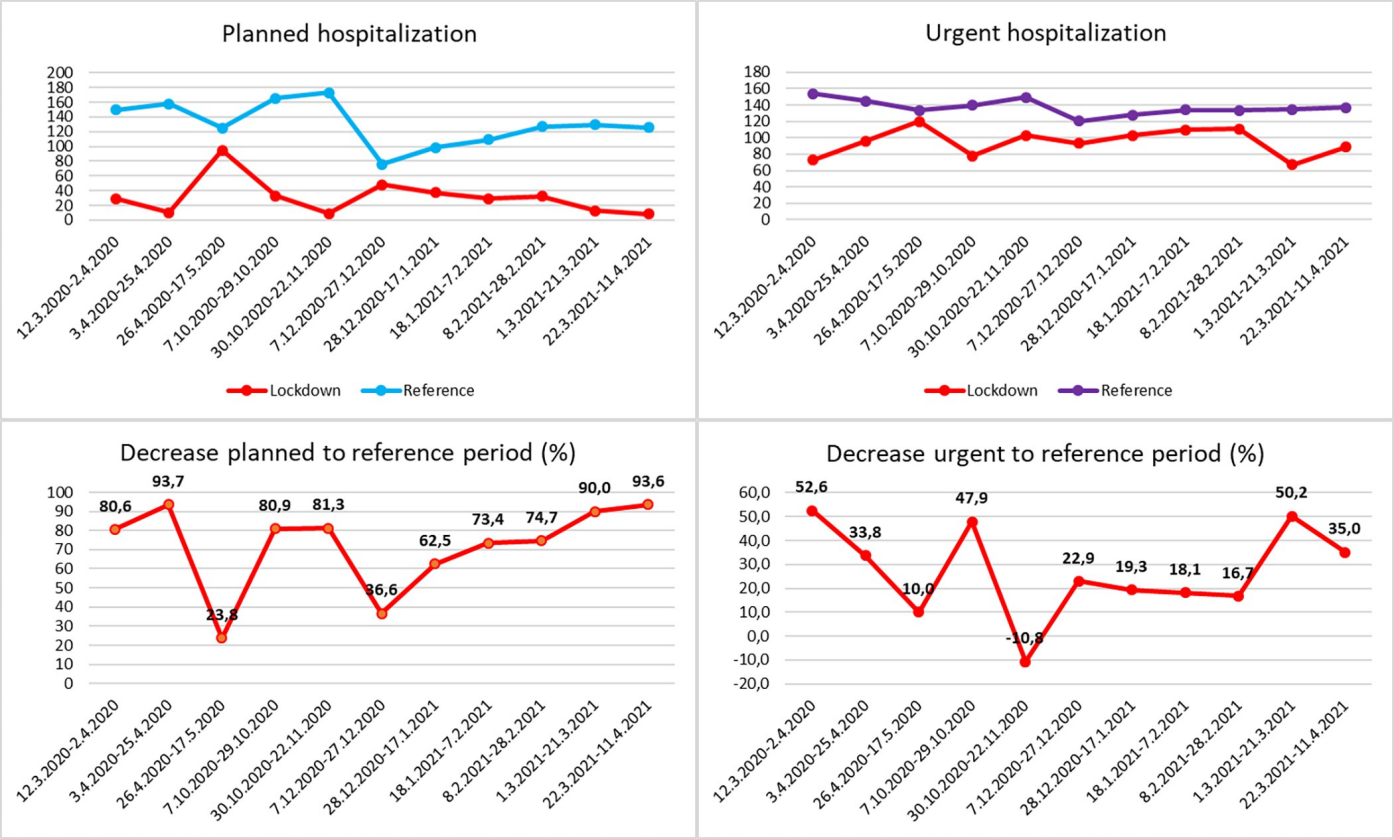
Overview of inpatients. Correlation of the number (y-axis) of elective (left) and acute inpatients (right) in the lockdown period and the reference period (in the upper part). In the case of the reference period, the average of the previous 3 years before the pandemic is calculated. Percentage decrease of inpatients in individual periods (in the bottom part). Negative values indicate an increase of inpatients.

**Fig 3:**
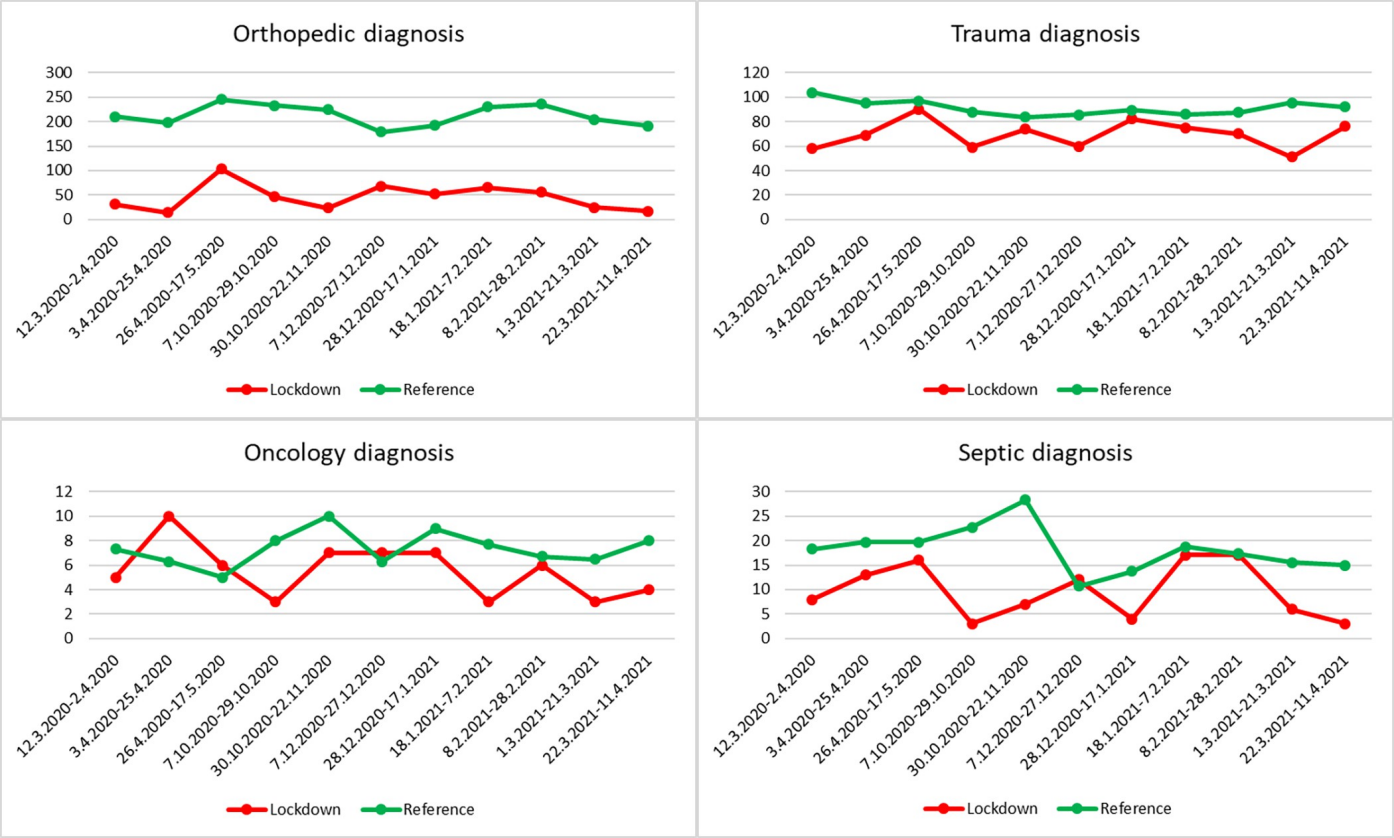
Admission diagnoses. Numbers (y-axis) of individual admission diagnoses during the lockdown period and reference period. For the reference period, the average of the previous 3 years before the pandemic is calculated.

**Fig 4:**
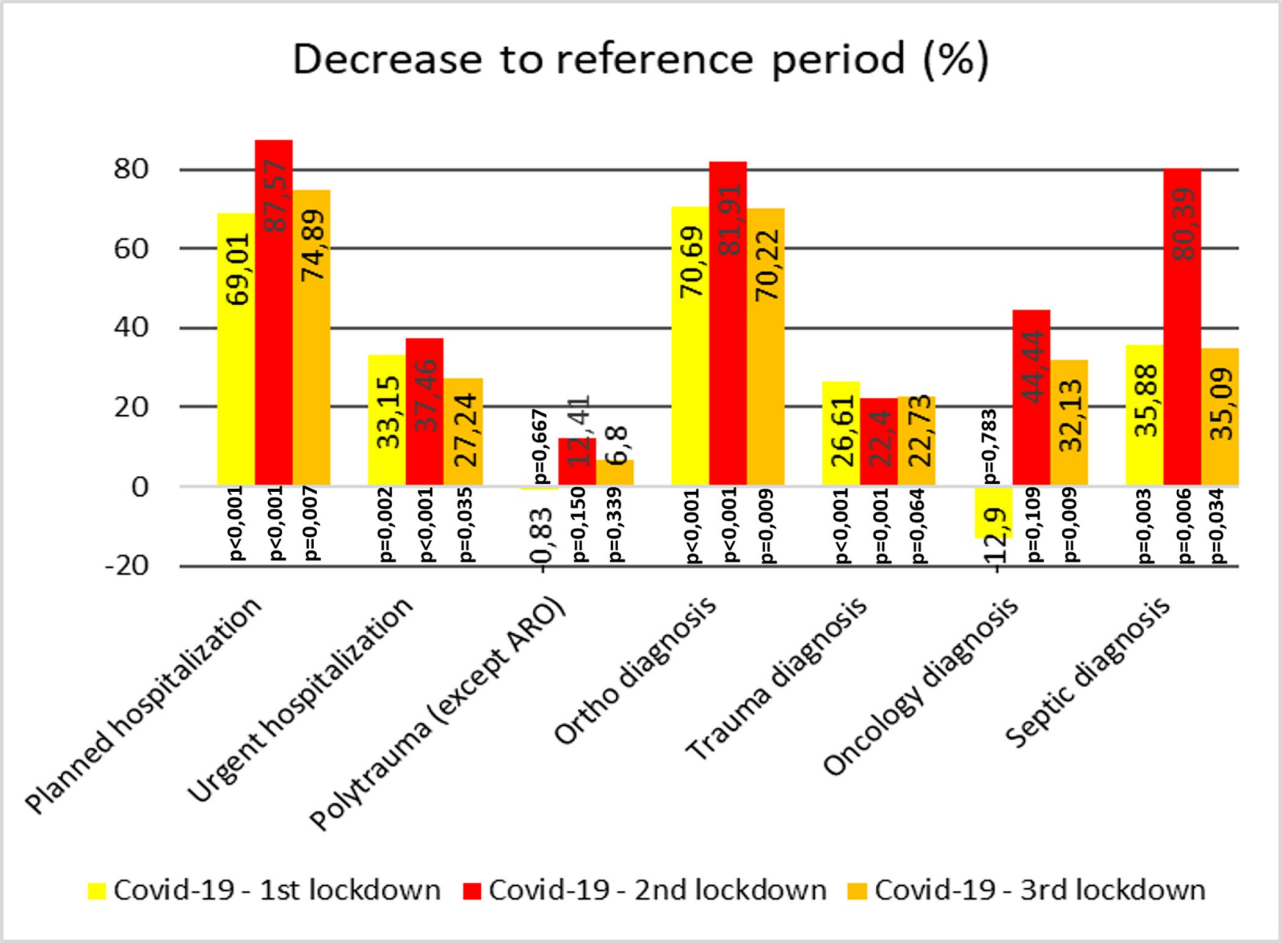
Decrease of admissions and lead diagnoses. Percentage decrease (y-axis) in the number of admissions and lead diagnoses in individual lockdown periods relative to the reference period. Negative values indicate an increase of inpatients. The p value indicates the statistical significance of the decrease.

Fig 2 shows the number of elective and acute inpatients during each monitored period. The number of inpatients in the reference period was calculated as the average for the last 3 years before the pandemic. Fig 3 shows the lead diagnosis on admission to the hospital. The number of elective surgeries (Fig 4) decreased by 69.01% (p <0.001), 87.57% (p <0.001), and 74.89% (p = 0.007) in the first, second and third lockdowns, respectively, during the lockdown period to the reference period of the previous 3 years. The number of acute surgeries (Fig 4) decreased by 33.15% (p = 0.002), 37.46% (p <0.001), and 27.24% (p = 0.034) in the first, second and third lockdowns, respectively, during the lockdown period compared with the reference period of the previous 3 years. The overall reduction in orthopaedic diagnoses was 70.69% (p <0.001), 81.91% (p <0.001), and 70.22% (p = 0.009) in the first, second and third lockdowns, respectively, compared with the reference period from the previous 3 years (Fig 4). The overall reduction in trauma diagnoses was 70.69% (p <0.001), 81.91% (p = 0.001), and 70.22% (p = 0.063) in the first, second and third lockdowns, respectively, compared with the reference period from the previous 3 years (Fig 4). The number of inpatients with oncological and septic diagnoses is summarised in Figs 3 and 4. Owing to the relatively small numbers, the percentage decrease (increase) in the number of inpatients can be misleading (p = 0.002 and p = 0.816 for septic and oncology patients, respectively). There were 1388 inpatients during the entire lockdown period, which is a total of 1556.7 patients lower in number than the reference period. This included a decrease of 1090.5 patients in elective admissions and 466.2 in acute admissions. Due to the length of the lockdown (a total of 240 days), there was a decrease of 6.49 inpatients per day (including weekends and holidays), including 4.54 for elective surgeries and 1.94 for trauma surgeries.

**Fig 5:**
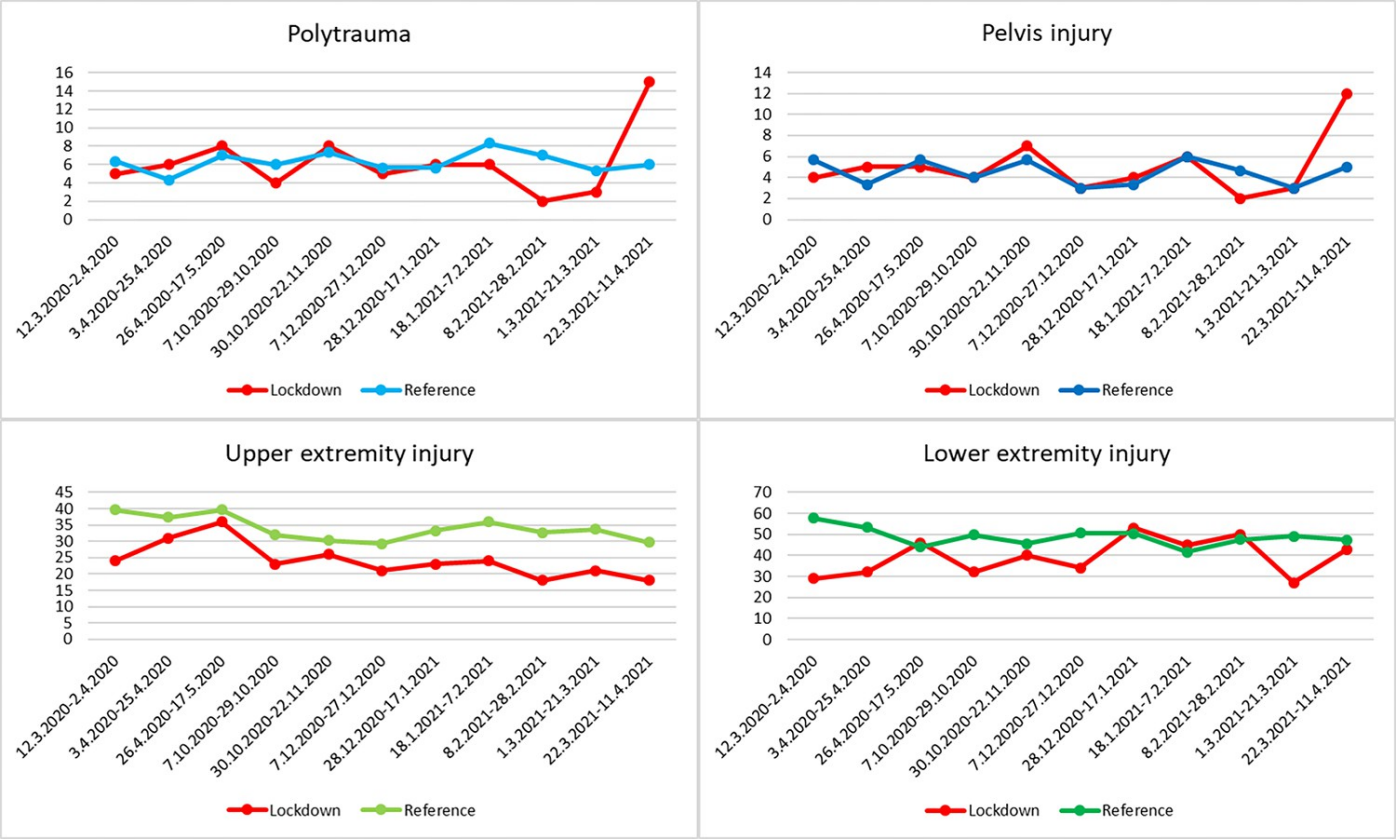
Overview of injuries. Number of individual injuries (y-axis) indicated for surgical treatment in the lockdown period and reference period.

**Fig 6:**
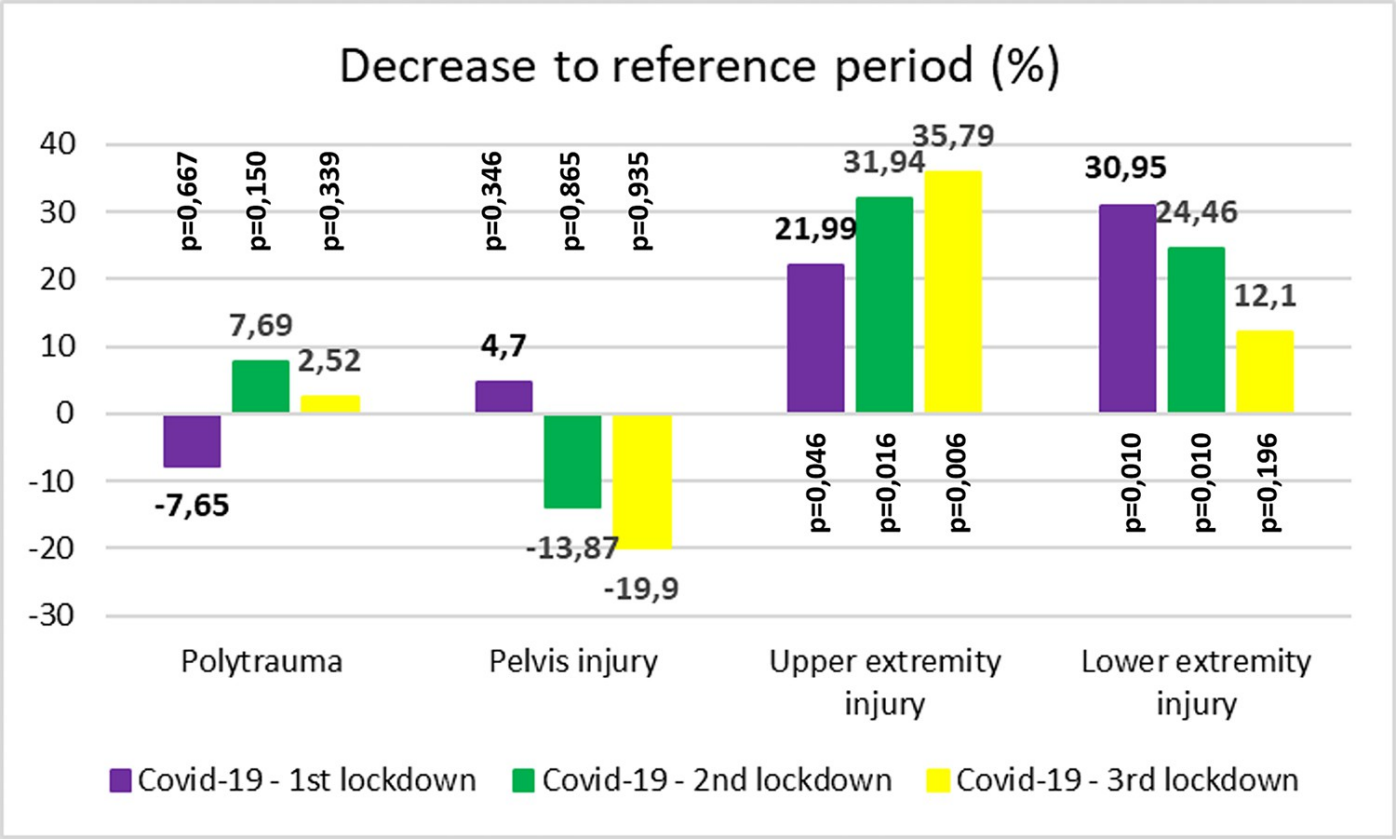
Decrease of injuries. Percentage decrease (y-axis) in the number of individual injuries in lockdown periods relative to the reference period. The p value indicates the statistical significance of the decrease. Negative values indicate an increase in the number of inpatients. The p value indicates the statistical significance of the decrease.

The number of injuries in individual anatomical localities did not differ statistically between polytrauma and pelvic injuries (p = 0.667 polytrauma, p = 0.347 pelvis) (Figs 5 and 6). For fractures in the upper extremity area (Figs 5 and 6) indicated for surgical treatment, the number of surgeries in the lockdown period decreased by 31.94 (p = 0.046) and 35.76% (p = 0.006) in the second and third lockdown, respectively, compared to the reference period of the previous 3 years. For fractures in the lower extremity area (Figs 5 and 6) indicated for surgical treatment, the number of surgeries in the lockdown period decreased by 30.95% (p = 0.010), 24.46% (p = 0.013), and 12.1% (p = 0.196) in the first, second, and third lockdown, respectively, compared to the reference period of the previous 3 years.

## Discussion

In the Czech Republic, which has a population of 10.7 million [12], during the first lockdown period (from 12^th^ March 2020 to 17^th^ May 2020), a total of 8300 patients tested positive for SARS-CoV-2 (in the capital city of Prague of 1.34 million inhabitants, 1945 patients.) [9, 13]. During the second lockdown (from 7^th^ October 2020 to 22^th^ November 2020) 40,0764 newly tested positive patients were registered in the Czech Republic (16,265 in Prague) [8, 9]. During the third lockdown (from 7^th^ December 2020 to 11^th^ April 2021), over a million patients (1,03,5192) (in Prague 51,819) tested positive in the Czech Republic [8, 9]. Regardless of the reasons for limited care, not only in Europe, but all over the world, there has been a significant delay in elective medical procedures.

Owing to the global pandemic, experiences and recommendations soon began to be published in orthopaedic journals [14, 15]. Orthopaedics, as a representative of elective care, was perhaps influenced by most of the disciplines in the field of elective surgery. Zhong compared the decline in the number of elective surgeries in the United States during the first wave of COVID-19 and showed a decrease in the number of elective surgeries by two-thirds and an increase in complications to 5.5% compared to 4.8% from the previous year [16]. The study also indicated that the number of readmissions after surgery was up to 27% [16]. Vasiliadis reported a decrease in orthopaedic surgery by 11.7%, decrease in trauma surgery by 8.9%, number of orthopaedic outpatients by 30.4%, and number of emergency outpatients by as much as 47.2% [17]. Similar restrictions on care have been reported in almost all orthopaedic facilities worldwide, either due to the reduced capacity of beds reserved for patients with COVID-19 or due to a lack of medical staff [2–7, 18]. Our study also showed a significant reduction in the care of orthopaedic and trauma outpatients (reduction in healthcare by 24–54%). We showed a reduction in the number of elective surgeries by 69–87%. In our opinion, the main reasons for the reduction in orthopaedic care were: i) reduced workplace capacity due to the conversion to Covid-19 units, ii) reduced performance due to the lack of medical staff, and iii) government bans on planned operations. The number of inpatients with COVID-19 in the orthopaedic department was 0, 42, and 121, in the first, second, and third lockdowns, respectively, which is approximately 6% (second lockdown) and 10% of all inpatients compared to the reference period. It is worth noting that the reduction in the number of trauma outpatients and trauma procedures may also be caused by restrictions on social life (especially restrictions on sports activities). Many studies have also pointed to limitations in the education of both medical students and young doctors (residents) [2–4].

The COVID-19 pandemic has implications for the economic side of individual workplaces, with a very gradual return to normal [1]. A significant economic downturn was also recorded in the private sector, where physician revenues were significantly reduced due to reduced care and, concurrent increased costs (especially for protective equipment, disinfectants, barrier measures, testing, etc.) [19]. The pandemic also had a significant economic impact on orthopaedic implant manufacturers [20]. Many smaller manufacturers faced financial problems due to the fall in the demand from orthopaedic surgery [20].

The COVID-19 pandemic has highlighted the lack of preparedness and shortcomings of individual healthcare systems worldwide. However, based on the experience gained, the development of organisational procedures has progressed. This includes, for example, the introduction of barrier procedures for patients with infectious diseases, including operating theatres (restrictions on the movement of people and materials, the availability of a sufficient number of protective equipment, etc.) [10, 21]. Work organisation protocols have been developed (working in shift teams, testing staff to eliminate the spread of disease, etc.) [11]. Based on experience, there has also been a technical improvement in the workplace for the provision of telemedicine services (distance control, consultations, etc.) or teaching methods (e-learning, online conferences, etc.) [2, 4, 18, 22].

The strengths of this study include unique data from the region that has never been published. It also contains data from the largest workplace in the country, which is very carefully processed. The limitations of the study include data from one workplace, which were evaluated retrospectively. Data on polytrauma, pelvic fractures, cancer, and sepsis included a small number of patients.

## Conclusion

This study showed a significant reduction in the healthcare of orthopaedic and trauma patients owing to the COVID-19 pandemic and emphasised the shortcomings of the healthcare system including both outpatient and inpatient care. Based on the results of this study, we recommend formulating specific organisational measures to mitigate the impact of the pandemic on orthopaedic healthcare (especially maximise the bed capacity of the workplace and work efficiency with regard to the safety of medical staff in individual medical facilities (intensive care unit, standard department, ambulance, and surgery theatre).

## Supporting information

S1 Dataset (XLSX)

S2 Dataset (XLSX)

S3 Dataset (XLSX)

S4 Dataset (XLSX)

S5 Dataset (XLSX)

S6 Dataset (XLSX)
